# Effect of Copper Sulphate Exposure on the Oxidative Stress, Gill Transcriptome and External Microbiota of Yellow Catfish, *Pelteobagrus fulvidraco*

**DOI:** 10.3390/antiox12061288

**Published:** 2023-06-16

**Authors:** Shun Zhou, Qiuhong Yang, Yi Song, Bo Cheng, Xiaohui Ai

**Affiliations:** 1 Yangtze River Fisheries Research Institute, Chinese Academy of Fishery Sciences, Wuhan 430223, China; 2 Hu Bei Province Engineering and Technology Research Center of Aquatic Product Quality and Safety, Wuhan 430223, China; 3 Chinese Academy of Fishery Sciences, No.150, Qingta West Road, Fengtai District, Beijing 100141, China; 4 Key Laboratory of Aquatic Product Quality and Safety Control, Ministry of Agriculture, No.150, Qingta West Road, Fengtai District, Beijing 100141, China

**Keywords:** copper sulphate, yellow catfish, oxidative stress, transcriptome, external microbiota

## Abstract

This study aimed to investigate the potential adverse effects of the practical application of copper sulfate on yellow catfish (*Pelteobagrus fulvidraco*) and to provide insights into the gill toxicity induced by copper sulphate. Yellow catfish were exposed to a conventional anthelmintic concentration of copper sulphate (0.7 mg/L) for seven days. Oxidative stress biomarkers, transcriptome, and external microbiota of gills were examined using enzymatic assays, RNA-sequencing, and 16S rDNA analysis, respectively. Copper sulphate exposure led to oxidative stress and immunosuppression in the gills, with increased levels of oxidative stress biomarkers and altered expression of immune-related differentially expressed genes (DEGs), such as IL-1β, IL4Rα, and CCL24. Key pathways involved in the response included cytokine–cytokine receptor interaction, NOD-like receptor signaling pathway, and Toll-like receptor signaling pathway. The 16S rDNA analysis revealed copper sulphate altered the diversity and composition of gill microbiota, as evidenced by a significant decrease in the abundance of Bacteroidotas and Bdellovibrionota and a significant increase in the abundance of Proteobacteria. Notably, a substantial 8.5-fold increase in the abundance of *Plesiomonas* was also observed at the genus level. Our findings demonstrated that copper sulphate induced oxidative stress, immunosuppression, and gill microflora dysbiosis in yellow catfish. These findings highlight the need for sustainable management practices and alternative therapeutic strategies in the aquaculture industry to mitigate the adverse effects of copper sulphate on fish and other aquatic organisms.

## 1. Introduction

Yellow catfish (*Pelteobagrus fulvidraco*) is a small freshwater fish species that dwells at the bottom of rivers and lakes, with a wide distribution in China and other Asian countries [1]. This species is extensively cultivated by farmers due to its high economic and nutritional value, as well as its strong adaptability to various environments. Due to the rapid advancement of modern breeding and culture techniques, the aquaculture of yellow catfish has flourished in recent years, reaching a production of 5.09 × 10^5^ tons in 2018 [2]. However, the occurrence of infectious diseases caused by parasitic protozoa, such as *Trichodina* sp., *Chilodonella* sp., and *Epistylis* sp., has resulted in significant deaths of yellow catfish, particularly juveniles, posing a severe risk to the yellow catfish aquaculture industry [3]. Currently, chemotherapy remains one of the primary strategies for treating protozoan infections in yellow catfish [4]. For instance, copper sulphate is widely used in aquaculture and has been proven to be effective in managing protozoan parasites [5].

As a commonly used antiparasitic agent, copper sulphate can be harmful to fish when used for the treatment of parasitic diseases, as it has the capacity to interfere with the normal physiological functions of fish, primarily by inhibiting enzyme activity and reducing the oxygen-carrying capacity of the hemoglobin [5]. Therefore, extensive research has been conducted to investigate the impact of copper sulphate on aquatic animals, including toxicity, histopathology, physiological changes, and immunological alterations [5,6,7,8]. For example, copper sulphate exposure has been shown to induce hyperemia, edema of primary and secondary epithelium, and hyperplasia of the gill epithelium in common carp (*Cyprinus carpio*) [9]. In tigris scraper (*Capoeta umbla*), exposure to copper sulphate has led to alterations in physiological and biochemical indicators such as superoxide dismutase (SOD), catalase (CAT), and glutathione reductase (GR) [8]. The utilization of copper sulphate to treat parasitic infections in aquaculture frequently relies on immersion techniques, which exposes the fish gills to this agent and makes them the most vulnerable organ. However, previous research on the toxicity of copper sulphate to fish gills has primarily been restricted to histopathology and the determination of some key physiological and biochemical indicators, thereby failing to offer a thorough comprehension of the gill toxicity induced via the practical application of copper sulphate in aquaculture [5,10].

Furthermore, the microbial community inhabiting the gills of fish is known to perform a range of vital functions, including nutrient uptake, metabolic regulation, and immune defense, thereby contributing to the overall health of the host [11,12]. The use of chemical agents in aquaculture can significantly perturb the diversity and community structure of bacteria in the gills of fish, which might pose a deleterious influence on the health of fish [13]. For instance, exposure to Chloramine-T has been shown to reshape the gill microbiome of Atlantic salmon (*Salmo salar*) by reducing bacterial load on gills [14]. Similarly, potassium permanganate exposure has resulted in disturbance of the gill microbial community of channel catfish (*Ictalurus puntactus*), thereby increasing host susceptibility to columnaris disease [15]. Nonetheless, the effect of the practical application of copper sulphate on the microbiota of fish gills remains unexplored.

To gain a comprehensive understanding of the adverse effects of the practical application of copper sulphate on the gills of fish, in this present study, yellow catfish were subjected to conventional anthelmintic concentrations of copper sulphate (0.7 mg/L) for 7 days. Subsequently, the oxidative stress biomarkers, including SOD, CAT, glutathione peroxidase (GSH-Px), as well as the contents of malondialdehyde (MDA), were measured in the gills, and the gene expression profiles and microbial communities in the gills of yellow catfish were also investigated.

## 2. Materials and Methods

### 2.1. Fish and Reagents

A batch of juvenile yellow catfish (average weight: 5.12 ± 0.89 g) was obtained from the fish breeding base of Huazhong Agricultural University. These fish were acclimated for a minimum of two weeks in 500 L aquariums containing 300 L of dechlorinated water in the laboratory. During the acclimatization period, these fish were fed with commercial feed twice daily, and the feces and debris were removed regularly, and one-third of the water was exchanged every three days. The water quality parameters were as follows: 21 ± 1 °C of temperature; 7.12 ± 0.45 for pH; 6.3 ± 0.4 mg/L for dissolved oxygen; less than 0.02 mg/L for ammonia nitrogen. Copper sulphate was purchased from J&K Scientific (Shanghai, China).

### 2.2. Experimental Design

A total of 120 healthy and swim-normally juvenile yellow catfish were randomly selected and assigned to two groups, each with three replicate plastic tanks containing 40 L of dechlorinated water and 20 yellow catfish. Subsequently, three tanks were selected and added with 0.7 mg/L copper sulphate, which is the recommended concentration for the treatment of parasites in aquaculture [16]. The remaining three tanks without drugs were used as the control group. The experimental environmental conditions were the same as during the acclimatization period. After one week of treatment, 15 yellow catfish from each group (five fish per tank in three replicate tanks) were randomly selected and anesthetized with 0.02% tricaine methanesulfonate (MS-222). Mucus samples were collected from the gills of the selected fish. Sterile swabs were used to scrape mucus from the right gill filaments between the first and second arch. To reduce heterogeneity, three swabs from different tanks within each experimental group were pooled into a single sample. After that, the gills were removed and separated into two parts, with one part used for enzyme activity assay and the other for RNA extraction. All samples were immediately frozen in liquid nitrogen and then stored at −80 °C for further experiments.

### 2.3. Determination of Antioxidant Enzyme Activities

The gill samples (three samples per treatment) were homogenized in a cold physiological saline solution (0.9% NaCl) at a ratio of 1:9 (*w*/*v*) using glass tissue homogenizers under ice-bath cooling. The homogenate was centrifuged at 4000 g for 20 min at 4 °C, and then the supernatant was collected for biochemical analysis. The enzyme activities of SOD, CAT, and GST, as well as the MDA level and protein content were determined using commercial kits (Nanjing Jiancheng Bioengineering Institute, Nanjing, China). SOD activity was assessed using the WST-1 (water-soluble tetrazolium-1) method at a wavelength of 450 nm [17]. One unit of SOD is defined as the quantity of the enzyme in a 20 μL sample solution that inhibits the reduction reaction of WST-1 with superoxide anion by 50%. CAT activity was determined by measuring the degradation of hydrogen peroxide at 405 nm [18]. One unit of CAT is defined as the amount of tissue protein that decomposes 1 µmol H_2_O_2_ and is expressed as U/mg protein. GST catalyzes the conjugation between 1-chloro-2,4 dinitrobenzene (CDNB) and GSH, and the activity of GST was determined by measuring the concentration of GSH at 412 nm [19]. One unit of GST is defined as the amount of enzyme necessary to reduce the GSH concentration by 1 µmol/L within one minute at 37 °C, excluding non-enzyme catalysis. MDA content was calculated based on the reaction of the generated substrate with thiobarbituric acid at 532 nm [20]. The protein concentration was quantified using bovine serum albumin solution as a standard following the Bradford method [21].

### 2.4. Transcriptomic Analysis

#### 2.4.1. RNA Extraction and Sequencing

The total RNA of gills samples (three samples per treatment) was extracted using TRlzol Reagent (Life Technologies, Carlsbad, CA, USA) according to the manufacturer’s instructions. The RNA concentration and quality were determined, and RNA libraries were prepared according to the conventional methods. The Illumina NovaSeq 6000 platform was used to perform paired-end sequencing with PE150 sequencing mode.

#### 2.4.2. Bioinformatic Analyses

The raw sequencing reads were trimmed of adapter sequences and low-quality regions, and clean reads were then mapped to the reference genome of yellow catfish (https://www.ncbi.nlm.nih.gov/genome/?term=Pelteobagrus_fulvidraco, accessed on 16 September 2022) using HISAT2 v2.2.1 software program. Gene expression levels were determined using the fragments per kilobase of transcript per million fragments mapped (FPKM) method, and differential expression analysis between treatment groups was conducted using DESeq2 v1.30.1. Differentially expressed genes (DEGs) were identified using the following criteria: |log 2 (Fold change)| > 1 and adjusted *p*-value < 0.05. Gene Ontology (GO) and Kyoto Encyclopedia of Genes and Genomes (KEGG) enrichment analysis of these DEGs was conducted using the ClusterProfiler packages in R software v4.1.0, with adjusted *p* < 0.05 as the cutoff.

#### 2.4.3. QRT-PCR Verification

To verify the reliability of the RNA-Seq results, the qPCR assays (three samples per treatment) were performed targeting nine DEGs identified in yellow catfish after exposure to copper sulphate. Primer pairs for these DEGs were designed and provided in Table S1. The qPCR assays were conducted using the QuantStudio TM 3 real-time PCR System with the same reaction system and thermal cycling conditions as described in our previous study [22]. The translation elongation factor 1-alpha 1 (*Elfa*) was used as an internal reference gene, and the 2^−ΔΔCt^ method was used to calculate the relative expression levels [23,24].

### 2.5. Microbiota Analysis

#### 2.5.1. DNA Extraction and Sequencing

The genomic DNA from the gills of yellow catfish (five samples per treatment) was extracted with the TGuide S96 Magnetic Soil/Stool DNA Kit (Tiangen Biotech (Beijing) Co., Ltd., Beijing, China), and the concentration of gDNA was also determined. The V3-V4 hypervariable region of the bacterial 16S rDNA gene was amplified, and the PCR reagents and cycling parameters are the same as previously described [25]. The PCR products were purified with Agencourt AMPure XP Beads (Beckman Coulter, Indianapolis, IN, USA), quantified using the Qubit dsDNA HS Assay Kit and Qubit 4.0 Fluorometer (Invitrogen, Thermo Fisher Scientific, Eugene, OR, USA), and sequenced on the Illumina NovaSeq 6000 platform with PE150 sequencing mode.

#### 2.5.2. Biodiversity Analysis

Clean reads were obtained via quality filtering and removal of primer sequences. Sequences with higher than 97% similarity were clustered into the same OTU (operational taxonomic unit) using USEARCH v10.0, and taxonomy annotation of the OTUs was performed using the SILVA database (release 138). Alpha diversity and beta diversity were calculated and displayed via the QIIME2 (release 2020.6) and R software v4.1.0, respectively. The differences in the relative abundances at the phylum and genus levels between the copper sulphate exposure group and control group were tested using one-way ANOVA, and a difference (*p* < 0.05) was considered significant. The functional profile of the gill microbiome was predicted using a PICRUSt2 analysis based on the KEGG annotations.

### 2.6. Statistical Analyses

The statistical analysis was conducted using SPSS 20.0 software (IBM, Armonk, NY, USA). Significant differences in the biochemical indexes, the gene expression levels, and the microbial alpha diversity and abundance between different groups were detected using a one-way analysis of variance (ANOVA) or an independent-sample t-test, and a *p* value less than 0.05 was considered significant.

## 3. Results

### 3.1. Behavior Observation and Antioxidant Enzyme Activity Analysis

During the entire duration of the experiment, no fish mortality occurred in the copper sulphate-exposed group and control group, and no significant behavioral abnormalities of the experimental fish were observed in both groups. The antioxidant enzyme activities and MDA content in the gills of yellow catfish after exposure to copper sulphate are shown in Table 1. Compared with the control group, the activity of SOD, CAT, and GSH-Px in the gill tissue of yellow catfish was significantly decreased, while the MDA content was significantly increased after exposure to copper sulphate.

### 3.2. Transcriptomic Analyses

#### 3.2.1. Transcriptome Statistics

Six cDNA libraries (CG1, CG2, CG3, TG1, TG2, and TG3) were constructed and sequenced from the gills of yellow catfish in the control and exposed groups, and the characteristics of these libraries are summarized in Table S2. A total of 43.42 Gb clean reads were obtained from the sequencing library, with the Q30 values greater than 93.98%. The mapping rate in each library ranged from 72.97% to 74.25%.

#### 3.2.2. Identification of Differentially Expressed Genes

**Figure 1:**
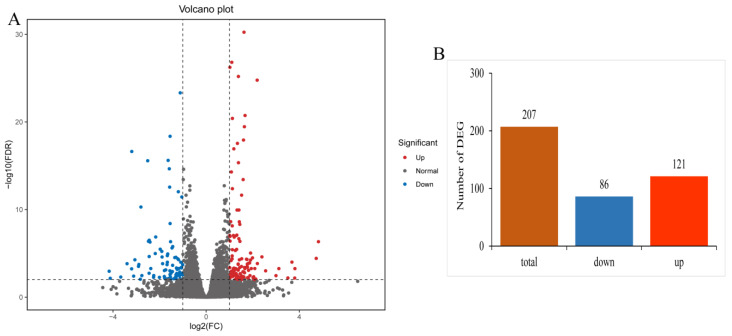
Summary of differentially expressed genes (DEGs) in gills of yellow catfish (*Pelteobagrus fulvidraco*) after exposure to 0.7 mg/L copper sulphate. (**A**) Volcano plot of DEGs. Red and blue dots represent significantly up-regulated and down-regulated DEGs, respectively. (**B**) Transcriptome analysis of the number and expression of DEGs.

**Figure 2:**
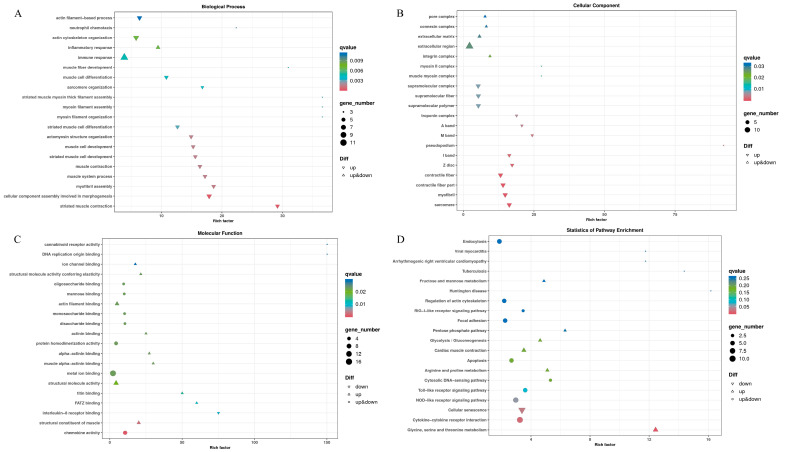
GO and KEGG enrichment analysis of the differentially expressed genes in the gills of yellow catfish (*Pelteobagrus fulvidraco*) after exposure to 0.7 mg/L copper sulphate. The vertical axis represents different GO terms (**A**–**C**) or pathways (**D**), and the horizontal axis represents rich factor.

**Figure 3:**
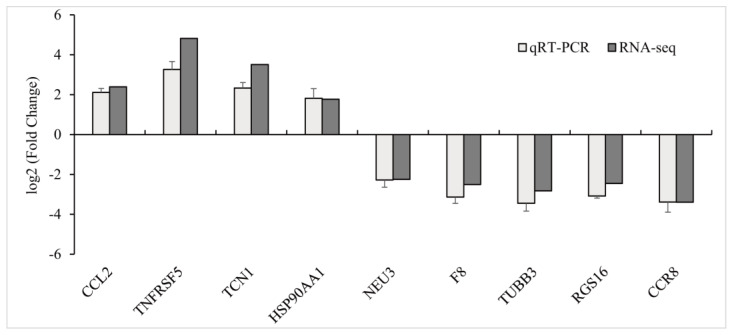
QRT−PCR verification of differentially expressed genes in the gills of yellow catfish (*Pelteobagrus fulvidraco*) after exposure to 0.7 mg/L copper sulphate. The *X*−axis displays 9 DEGs, and the *Y*−axis represents relative fold change. The data are expressed as the means ± SD (n = 3).

A total of 207 differentially expressed genes (DEGs) were identified between the control group and copper sulphate exposure group via differential expression analysis, of which 121 were up-regulated and 87 were down-regulated (Figure 1). Subsequently, these DEGs were subjected to GO and KEGG enrichment analysis. GO analysis indicated that 168 DEGs were successfully assigned into three main groups, including biological process, cellular component, and molecular function. In the biological process category, striated muscle contraction (GO:0006941) and cellular component assembly involved in morphogenesis (GO:0010927) were the most enriched GO terms. The most enriched GO terms in the cellular component category were sarcomere (GO:0030017) and myofibril (GO:0030016). In the molecular function category, the most enriched GO terms were chemokine activity (GO:0008009) and structural constituent of muscle (GO:0008307) (Figure 2). The KEGG pathway enrichment analysis demonstrated that 163 DEGs were enriched in 76 KEGG pathways, and the most significantly enriched pathways were glycine, serine, and threonine metabolism (ko00260); cytokine–cytokine receptor interaction (ko04060); and cellular senescence (ko04218) (Figure 3). Moreover, some immune-related pathways were also enriched, such as NOD-like receptor signaling pathway (ko04621), Toll-like receptor signaling pathway (ko04620), C-type lectin receptor signaling pathway (ko04625), and intestinal immune network for IgA production (ko04672). Several immune-related DEGs in the gills of yellow catfish after exposure to copper sulphate were listed in Table S3.

#### 3.2.3. QRT-PCR Verification

To verify the results obtained via RNA sequencing, nine DEGs were selected and subjected to qPCR analysis. In general, the gene transcription levels obtained from the qPCR assays exhibited a similar pattern and degree of alterations in comparison to the transcriptomic results, hereby providing further support for the reliability of the RNA-seq data (Figure 3).

### 3.3. Microbiota Analysis

#### 3.3.1. Sequencing Analysis and Taxonomic Annotation

**Figure 4:**
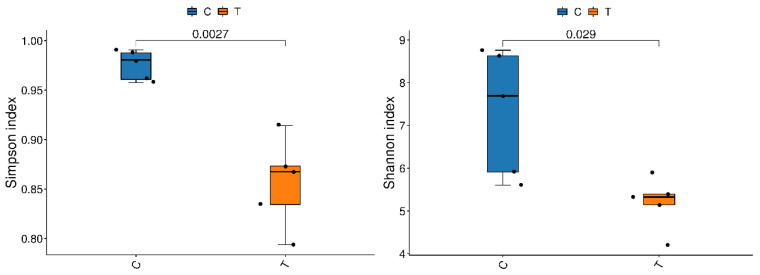
Simpson index and Shannon index of bacterial communities in the gills of yellow catfish (*Pelteobagrus fulvidraco*) after exposure to 0.7 mg/L copper sulphate. C: 0 mg/L copper sulphate; T: 0.7 mg/L copper sulphate.

A total of 799,446 clean reads were obtained from the gills of yellow catfish, with the number of clean reads ranging from 79,578 to 80,380 in different samples (Table S4). Rarefaction curves of sequences indicated that the sequencing depth was sufficient and the sequencing data were suitable for further analysis (Figures S1 and S3). These sequences were then subjected to clustering analysis, resulting in the identification of 7718 OTUs. Among these, 331 OTUs were shared by the control group and copper sulphate exposure group. Alpha-diversity indices of the gill microbiota of yellow catfish after exposure to copper sulphate are listed in Table S5. Statistical analysis indicated that the Chao, ACE, and PD whole tree indices decreased in the copper sulphate exposure group, but there was no statistically significant difference (*p* > 0.05). Additionally, significant decreases in the Simpson and Shannon indicators were noted in the gills of yellow catfish that were subjected to copper sulphate exposure (*p* < 0.05) (Figure 4).

#### 3.3.2. Microbial Composition

**Figure 5:**
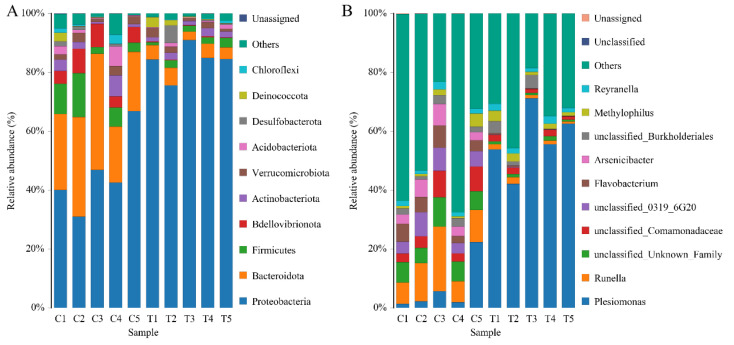
Relative abundances of dominant microbial phyla (**A**) and genera (**B**) in the gills of yellow catfish (*Pelteobagrus fulvidraco*) after exposure to 0.7 mg/L copper sulphate. C: 0 mg/L copper sulphate; T: 0.7 mg/L copper sulphate.

To identify the alterations of the gill microbial community of yellow catfish after exposure to copper sulphate, the composition and relative abundance of the microbiota from the gills were analyzed. The most dominant phyla of the microbial community were Proteobacteria, Bacteroidotas, Firmicutes, Bdellovibrionota, and Actinobacteriota (Figure 5A and Figure S2). These five phyla accounted for more than 78% of the total sequences in all samples. The abundance of Bacteroidotas and Bdellovibrionota was significantly decreased, while the abundance of Proteobacteria was significantly increased in the gills of yellow catfish after exposure to 0.7 mg/L copper sulphate (*p* < 0.05) (Table 2). The ratio of Bacteroidas/Firmicutes was decreased in the copper sulphate exposure group (6.37 ± 2.94) compared to the control group (2.47 ± 0.68) but statistically non-significant (*p* > 0.05).

**Figure 6:**
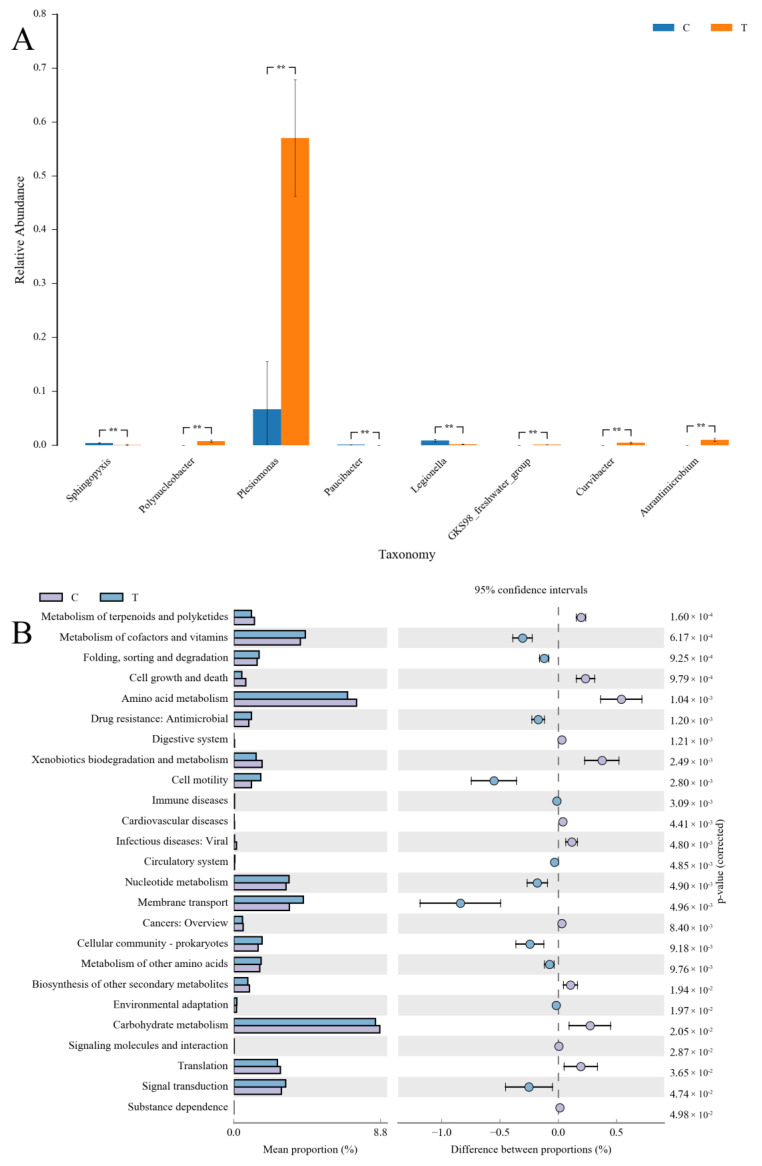
(**A**) The abundance differences in microbial taxa of yellow catfish (*Pelteobagrus fulvidraco*) between the control group and copper sulphate exposure group at the genus level, ** *p* < 0.01; (**B**) KEGG analysis identified various enriched pathways between the control group and copper sulphate exposure group. C: 0 mg/L copper sulphate; T: 0.7 mg/L copper sulphate.

At the genus level, the dominant bacteria in the gills of yellow catfish were *Plesiomonas*, *Runella*, *Flavobacterium*, *Arsenicibacter*, and *Methylophilus* (Figure 5B). In the copper sulphate exposure group, the top eight dominant genera with significant differences (*p* < 0.05) in abundance were *Plesiomonas*, *Legionella*, *Sphingopyxis*, *Paucibacter*, *Polynucleobacter*, *Curvibacter*, GKS98 freshwater group, and *Aurantimicrobium* (Figure 6A). Notably, a significant 8.5-fold increase in the abundance of *Plesiomonas* was observed in the copper sulphate exposure group compared to the control group. In the KEGG analysis, carbohydrate metabolism and amino acid metabolism were the most enriched categories of gill microbial function in the control group and copper sulphate exposure group.

## 4. Discussion

Copper sulphate is a widely used chemical agent in aquaculture for the treatment of infectious diseases caused by parasitic protozoa, such as *Cryptobia* sp., *Trichodina* sp., and *Chilodonella* sp., as well as crustacean diseases, such as *Sinergasilus* sp. [26,27]. Copper sulphate is usually applied in therapeutic baths, and the fish gills are, thus, one of the most vulnerable organs due to direct contact with the external environment. Despite this, the gill toxicity inducted via the practical application of copper sulfate in aquaculture has been rarely explored. In this present study, the changes in the molecular indices of oxidative stress, transcriptomic profile, and microbial communities in the gills of yellow catfish were investigated following copper sulphate exposure, aiming to reveal the potential adverse effects of the practical application of copper sulfate in aquaculture.

The antioxidant system is one of the primary defense lines for the host to resist external environmental stress [28]. This system is able to prevent oxidative damage to cellular components by activating enzyme systems such as SOD and CAT [29]. In the current study, the activity of SOD, CAT, and GSH-Px was significantly decreased, and the MDA content was significantly increased after exposure to copper sulphate. MDA serves primarily as an indicator of lipid peroxidation. However, in this study, MDA levels were measured relative to tissue protein levels rather than fat content, which may not accurately reflect meaningful parameters of oxidative stress. Nevertheless, these results indicated that exposure to 0.7 mg/L copper sulphate can significantly inhibit antioxidant enzyme activity in the gills of yellow catfish. These findings of this study are in agreement with those of a previous investigation, which has shown that exposure to 0.1–1.5 mg/L copper sulphate resulted in increased lipid peroxidation and decreased levels of SOD, CAT, and GSH-Px activities in the liver of goldfish (*Carassius auratus*) [30]. Similarly, reduced GSH-Px and CAT activity and increased MDA content were also observed in the liver and gills of rainbow trout (*Oncorhynchus mykiss*) after exposure to 5 μg/L copper sulphate [31]. On the contrary, 25–200 μg/L of copper ions induced a rapid and transient increase in SOD, CAT, and GSH-Px activity in the three-spined stickleback (*Gasterosteus aculeatus*) [32]. The activity of antioxidant enzymes also showed a trend of continuous increase in the liver and kidney of snake-headed murrel (*Channa punctatus*) after exposure to various sublethal concentrations of copper sulphate [33]. The divergent trends observed in the antioxidant enzyme activity induced by copper sulphate across various fish species may be attributed to differences in exposure dosage and time, the specific tissues examined, and the varying tolerance to copper sulphate. Fish species display differing levels of tolerance to copper sulphate, with some being highly susceptible and experiencing mortality even at low concentrations, while others exhibit greater tolerance. For instance, the 72 h-LD_50_ of copper sulphate was found to be 2.01 mg/L in grass carp (*Ctenopharyngodon idella*), whereas, in Nile tilapia (*Oreochromis niloticus*), it was determined to be significantly higher at 40.6 mg/L [34,35]. Moreover, several environmental factors, such as pH and water hardness and alkalinity, can affect the forms of copper ions present in the aquatic environment, ultimately determining its bioavailability to organisms and further affecting their toxicity [5,7]. Overall, our findings demonstrated that exposure to 0.7 mg/L copper sulphate for seven days has potential to induce oxidative stress in the gills of yellow catfish.

The immune system of fish plays a crucial role in maintaining their health and is vulnerable to external environmental stressors [36,37]. In this trial, the immune response was significantly enriched in the GO analysis and several immune-related pathways, such as Cytokine-cytokine receptor interaction, NOD-like receptor signaling pathway, and Toll-like receptor signaling pathway were also enriched. Specifically, the expression levels of many immune-related DEGs, such as interleukin-1 beta-like (IL-1β), macrophage mannose receptor 1-like (MRC1L), and CD209 antigen-like protein D isoform X1 (CD209D), were significantly downregulated in the gills of yellow catfish following copper sulphate exposure. IL-1β plays a critical role in regulating the immune response by stimulating the production of other cytokines and promoting the differentiation and proliferation of lymphocytes [38]. MRC1L and CD209D are important immune molecules that exhibit a variety of immune functions, including pathogen recognition and clearance, regulation of immune response, and promotion of immune cell activation [39,40]. The downregulation of these genes indicated that recommended anthelmintic concentrations of copper sulphate (0.7 mg/L) can perturb the host’s immune system. Similarly, previous studies have also reported that copper exposure could lead to immunosuppression in the olfactory mucosa of rainbow trout and the intestines of juvenile orange-spotted grouper (*Epinephelus coioides*) [41,42]. Moreover, in this current study, inflammatory-related GO terms, including inflammatory response, response to interleukin-1, and response to tumor necrosis factor, were also significantly enriched. Several inflammatory-related DEGs, such as interleukin-4 receptor subunit alpha-like (IL4Rα), C-X-C motif chemokine 3-like isoform X1 (CXCL3), and C-C motif chemokine 24-like (CCL24), showed significant fluctuations in the copper sulphate exposure group compared with the control group. Additionally, IL-1β, a pro-inflammatory factor mentioned above, was significantly downregulated. Similarly, 0.25 mg/L copper exposure significantly decreased the gene expression of IL-1β, IL-10, and TNF-α in the gills of common carp [43]. These results suggested that copper sulphate exposure induced immunosuppression in the gills of yellow catfish.

The gill microbiome of fish is essential in protecting the host organism from bacterial infection by either competing with pathogens for space or nutrients, producing antagonistic compounds, or interacting with the host’s immune system [44]. Due to direct exposure to the external environment, the gill microbial community is particularly vulnerable to external environmental stressors [45]. Pathogen infections and drug treatments disrupt the gill microbial community, which may, in turn, increase the host’s susceptibility to other opportunistic pathogens [13,14,15,46]. For instance, exposure to potassium permanganate and chloramine-T resulted in significant disturbances to the external microbiomes of channel catfish and Atlantic salmon, respectively [14,15]. In this study, noteworthy declines in the Simpson and Shannon indicators were observed in the gill of yellow catfish after exposure to copper sulphate, suggesting an imbalance of the gill microbial community inducted by copper sulphate.

In terms of microbial composition in the gill of yellow catfish, the most dominant phyla observed were Proteobacteria, Bacteroidotas, and Firmicutes, which is consistent with the results of comparable studies on other fish species [47,48,49]. In this trial, the relative abundance of Proteobacteria was significantly increased after exposure to copper sulphate. Similar results have been reposted in the gill and skin of common carp following exposure to povidone iodine [13]. Proteobacteria is generally considered to be the most diverse and adaptable of the major phyla in the intestinal flora, exhibiting a wide range of metabolic and physiological characteristics [50]. The relative abundance of Proteobacteria is easily influenced by various factors, including oxygen contents, drug treatment, genetic susceptibility, and enteritis. The increased abundance of Proteobacteria is a potential diagnostic signature of metabolic and immune dysbiosis and risk of disease [50,51]. Therefore, although the exact physiological role of Proteobacteria in the gills of yellow catfish remains unclear, future studies should investigate whether the observed increase in their abundance is associated with the immune disorder following exposure to copper sulphate. In this study, the increase in Proteobacteria abundance was primarily attributed to the proliferation of *Plesiomonas*, a genus of Gram-negative bacteria frequently found in aquatic environments [52]. The genus *Plesiomonas* consists of only one species, namely *P. shigelloides*, which is recognized as a potential pathogen for humans and animals [52]. This species has been proven to be highly pathogenic for cichlid ornamental fish (*Pseudotropheus socolofi*) and silver carp (*Hypophthalmichthys molitrix*), and its infections can result in massive host mortality [53,54]. Furthermore, the abundance of Bdellovibrionota was also notably reduced. The phylum Bdellovibrionota consists of obligate predators that have the capability to attack and destroy other bacteria, including pathogens [55]. Although there is limited research on the role of Bdellovibrionota in the external microbiomes of fish, available evidence suggests that these bacteria may have a protective effect against harmful bacterial pathogens. For instance, *Bdellovibrio*, a genus of the phylum Bdellovibrionota, worked in conjunction with the host immune system to effectively treat *Shigella* infection and increase the survival of zebrafish (*Danio rerio*) [56]. Thus, exposure to copper sulphate might lead to an elevation of potentially pathogenic bacteria and a decline in the levels of potentially beneficial bacteria in the gill microenvironment of yellow catfish, which could be detrimental to the host’s health.

In addition, the abundance of Bacteroidete and Firmicutes was found to be decreased after copper sulphate exposure. Similarly, a previous study also showed that povidone-iodine exposure also induced a decrease in the abundance of Bacteroidotas and Firmicutes in koi carp (*C. carpio*) [13]. A previous study also indicated that copper sulphate treatment led to a notable reduction in the abundance of Bacteroidotas on the external microbiota of adult common snook (*Centropomus undecimalis*), whereas the abundance of Firmicutes increased [57]. Bacteroidete and Firmicutes are crucial in polysaccharide catabolism. Specifically, Bacteroidetes are responsible for decomposing complex polysaccharides and generating different enzymes, whereas Firmicutes ferment complex carbohydrates and produce short-chain fatty acids [58]. The relative abundance of these two phyla has been extensively investigated as indicators for gut health and dysbiosis [59]. Consequently, the results demonstrated that copper sulphate exposure had the potential to disrupt carbohydrate metabolism in yellow catfish by modifying the composition of gill microorganisms, as evidenced by a reduced abundance of Bacteroidete and Firmicutes, along with a lower Bacteroidete/Firmicutes ratio. The findings were substantiated via PICRUSt2 analysis, which revealed the inhibition of carbohydrate metabolism due to copper sulphate treatment.

## 5. Conclusions

In summary, the results of this study provide novel insights into the gill toxicity induced via the practical application of copper sulfate in aquaculture using biochemical assays, transcriptome, and microbiome analyses. Acute exposure to conventional anthelmintic concentrations of copper sulphate can induce oxidative stress and disturb the immune system in the gills of yellow catfish. Additionally, copper sulphate treatment also caused an imbalance of the gill microbial community and resulted in an elevation of potentially pathogenic bacteria and a decline in the levels of potentially beneficial bacteria in the gill microenvironment of yellow catfish, which could be detrimental to the host’s health. Overall, the findings presented in this study have important implications for our understanding of the gill toxicity of copper sulphate to fish and highlight the need for developing strategies to mitigate the adverse effects of copper sulphate on fish and other aquatic organisms.

## Figures and Tables

**Table 1:** Effect of copper sulphate exposure on superoxide dismutase (SOD), catalase (CAT), Glutathione peroxidase (GSH-Px), as well as the contents of malondialdehyde (MDA) in the gill tissue of yellow catfish (*Pelteobagrus fulvidraco*). * *p* < 0.05 (N = 3, mean ± SD).

Treatment	SOD (U/mg Protein)	CAT (U/mg Protein)	GSH-Px (U/mg Protein)	MDA (nmol/mg Protein)
Control group	55.45 ± 3.19	6.84 ± 0.53	6.63 ± 0.7	5.91 ± 1.51
Exposed group	39.19 ± 3.15 *	4.8 ± 0.6 *	4.69 ± 0.68 *	10.94 ± 1.36 *

**Table 2:** The microbial composition (mean ± SE, N = 5) in the gills of yellow catfish (*Pelteobagrus fulvidraco*) after exposure to copper sulphate at the phylum level, * *p* < 0.05.

Phylum	Relative Abundance (%)	
	0 mg/L Copper Sulphate	0.7 mg/L Copper Sulphate
Bacteroidotas	27.64 ± 3.96	4.51 ± 0.51 *
Bdellovibrionota	5.93 ± 0.92	0.26 ± 0.04 *
Proteobacteria	45.46 ± 5.93	84.07 ± 2.46 *
Firmicutes	7.4 ± 2.35	2.16 ± 5.83

## Data Availability

Raw RNA-seq data and 16S rRNA data have been deposited in the NCBI Sequence Read Archive (SRA) database with the accession numbers PRJNA955189 and PRJNA955022, respectively. The datasets used and analyzed during this current study are available from the corresponding author upon reasonable request.

## Supplementary Materials

The following supporting information can be downloaded at: https://www.mdpi.com/article/10.3390/antiox12061288/s1, Table S1: The primers used in the quantitative PCR analysis; Table S2: Summary statistics of the transcriptome sequences. CG: 0 mg/L copper sulphate; TG: 0.7 mg/L copper sulphate; Table S3: Representative immune-related DEGs in yellow catfish after exposure to copper sulphate; Table S4: Characteristics of 16S rRNA sequences of yellow catfish (*Pelteobagrus fulvidraco*) gills. C: 0 mg/L copper sulphate; T: 0.7 mg/L copper sulphate; Figure S1: Rarefaction curves of yellow catfish (*Pelteobagrus fulvidraco*) gills tissue samples. C1–C5: control group; T1–T5: 0.7 mg/L copper sulphate exposed group; Table S5: Richness and diversity indices of bacterial communities for all gill tissue samples. C: 0 mg/L copper sulphate; T: 0.7 mg/L copper sulphate; Figure S2. (Left) Hierarchical cluster analysis based on the unweighted pair group method with arithmetic mean (UPGMA); (Right) Relative abundances of dominant microbial genera. C: copper sulphate; T: 0.7 mg/L copper sulphate; Figure S3: Principal coordinates analysis (PCoA) of the microbial communities. C: 0 mg/L copper sulphate; T: 0.7 mg/L copper sulphate.
