# Supplementary material for: Effect of Copper Sulphate Exposure on the Oxidative Stress, Gill Transcriptome and External Microbiota of Yellow Catfish, Pelteobagrus fulvidraco
Source: Antioxidants (Basel). 2023 Jun 16;12(6):1288. doi: 10.3390/antiox12061288 (PMC10295726; doi:10.3390/antiox12061288)
Supplement: Supplementary file 1 [file antioxidants-12-01288-s001.zip › Supporting Information.pdf]

Supporting Information

Effect of copper sulphate exposure on the oxidative stress, gill transcriptome and external microbiota of yellow catfish, *Pelteobagrus fulvidraco*

Shun Zhou [1,2], Qiuhong Yang [1,2], Yi Song [3,4], Bo Cheng [3,4,*] and Xiaohui Ai [1,2,*]

[1] Yangtze River Fisheries Research Institute, Chinese Academy of Fishery Sciences, Wuhan 430223, China
[2] Hu Bei Province Engineering and Technology Research Center of Aquatic Product Quality and Safety, Wuhan 430223, China
[3] Chinese Academy of Fishery Sciences, No.150, Qingta West Road, Fengtai District, Beijing 100141, China
[4] Key Laboratory of Aquatic Product Quality and Safety Control, Ministry of Agriculture, No.150, Qingta West Road, Fengtai District, Beijing 100141, China
* Correspondence: chengb@cafs.ac.cn (B.C.); aixh@yfi.ac.cn (X.A.)

Table S1. The primers used in the quantitative PCR analysis.

Name	Forward primers (5’-3’)	Reverse primers (5’-3’)	Accession no.
CCL2	CCTGCCTTCAGTCCTTCACAAT	ACTGATCTGGTACATTTCTCTTCTC	OR062247
TNFRSF5	TCTCCTGAAGGTGTGAACTGC	AGGTATTTGACCAGATGCCATGA	OR062248
TCN1	AACTACGGCCCATTCCTTGT	GCTGTGTCAAATGGGTTTGGT	OR062249
HSP90AA1	TCAGTAGAGGGCCAACTGGA	AGACTCTGCGCACATAGAGC	OR062250
NEU3	GCACCATGAATCCGTGTCCT	CTTCCCTGTCCTTATCTGGTGT	OR062251
F8	TTACGTCGTCAAGCCCACTC	GCGCTGCCTTCTTGGTTAAG	OR062252
TUBB3	CCAACAGTACCGCGCTCTTA	CCGTGCTGTTGCCGATAAAG	OR062253
RGS16	GGATGCGCTCAAGTGGAAAG	CAGACATCAGGAACACCCGA	OR062254
CCR8	GCTGGGCAATGGTTTGGTTC	ACTTGCATCCCATCTTGCCA	OR062255
Elfa	GTCTGGAGATGCTGCCATTG	AGCCTTCTTCTCAACGCTCT	KU886307

Abbreviations: CCL2, C-C motif chemokine 2-like; TNFRSF5, tumor necrosis factor receptor superfamily member 5-like; TCN1, transcobalamin-1-like isoform X1; HSP90AA1, heat shock protein HSP 90-alpha 1; NEU3, sialidase-3-like; F8, coagulation factor VIII-like; TUBB3, tubulin beta-3 chain; RGS16, regulator of G-protein signaling 16-like; CCR8, C-C chemokine receptor type 8-like; Elfa, translation elongation factor 1-alpha 1.

Table S2. Summary statistics of the transcriptome sequences. CG: 0 mg/L copper sulphate; TG: 0.7 mg/L copper sulphate.

Sample	Clean reads	Clean bases	GC content (%)	Q30 (%)	Total Mapped reads (ratio)	Uniquely mapped reads (ratio)	Multiple mapped reads (ratio)
CG1	22,813,750	6,804,749,242	46.05%	94.79%	33,728,222 (73.92%)	31,900,936 (69.92%)	1,827,286 (4.00%)
CG2	23,041,240	6,879,498,494	45.78%	93.98%	33,625,353 (72.97%)	31,965,235 (69.37%)	1,660,118 (3.60%)
CG3	26,233,930	7,832,889,386	45.95%	94.20%	38,687,544 (73.74%)	36,747,590 (70.04%)	1,939,954 (3.70%)
TG1	25,036,723	7,459,653,852	45.98%	94.80%	37,166,231 (74.22%)	35,254,330 (70.41%)	1,911,901 (3.82%)
TG2	24,412,195	7,286,585,586	45.90%	94.74%	36,188,760 (74.12%)	34,365,123 (70.39%)	1,823,637 (3.74%)
TG3	23,959,591	7,154,763,972	45.80%	94.41%	35,487,906 (74.06%)	33,442,507 (69.79%)	2,045,399 (4.27%)

Table S3. Representative immune-related DEGs in yellow catfish after exposure to copper sulphate.

Gene ID	Gene description	Change	log2FoldChange	FDR
**Cytokine-cytokine receptor interaction**				
gene_14548	C-X-C chemokine receptor type 4-like	up	1.767553	0.000166
Tachysurus_fulvidraco_newGene_5764	C-C motif chemokine 24-like	down	-1.79552	0.004344
gene_8371	C-C chemokine receptor type 8-like	down	-3.39556	0.000151
gene_20740	interleukin-13 receptor subunit alpha-1-like isoform X1	down	-1.13274	0.001362
gene_1927	C-C motif chemokine 2-like	up	2.394937	2.52E-05
gene_11637	tumor necrosis factor receptor superfamily member 5-like	up	4.822969	4.64E-07
gene_25805	interleukin-4 receptor subunit alpha-like	down	-1.29709	0.002819
gene_11027	regakine-1-like	down	-1.97259	3.35E-06
**NOD-like receptor signaling pathway**				
gene_1763	permeability factor 2-like	down	-3.19332	2.35E-17
gene_8551	thioredoxin-interacting protein-like	up	1.194068	0.000484
gene_2649	heat shock protein HSP 90-alpha 1	up	1.772629	0.000385
gene_17388	arrestin domain-containing protein 2-like	up	1.605294	1.16E-18
gene_25109	interferon-induced very large GTPase 1-like	down	-1.46529	2.43E-06
gene_1764	C-X-C motif chemokine 3-like isoform X1	down	-3.05904	5.42E-05
gene_24531	thioredoxin-interacting protein-like	up	1.344409	2.79E-18
**Apoptosis**				
gene_1241	calpain-2 catalytic subunit-like	down	-3.66568	0.005042
gene_1237	calpain-2 catalytic subunit-like	down	-1.7344	0.000764
gene_21018	interferon-induced protein 44-like isoform X1	down	-1.26364	0.00199
gene_20615	interferon-induced protein 44-like isoform X1	down	-1.27477	0.000468
gene_9008	perforin-1-like isoform X1	up	1.028376	1.16E-07
**MAPK signaling pathway**				
gene_7728	ephrin-A1-like	up	1.19025	1.17E-17
Tachysurus_fulvidraco_newGene_1639	uncharacterized protein LOC113546666	down	-1.66612	1.83E-05
gene_11717	dihydropyridine-sensitive L-type skeletal muscle calcium channel subunit alpha-1-like	up	1.605011	0.001332

**Phagosome**				
gene_1494	macrophage mannose receptor 1-like	down	-1.17446	0.000542
gene_19386	BOLA class I histocompatibility antigen, alpha chain BL3-7-like	down	-1.67818	0.000194
gene_5057	tubulin beta-3 chain	down	-2.825	0.009038
gene_19380	BOLA class I histocompatibility antigen, alpha chain BL3-7-like	down	-1.28525	0.007084
**C-type lectin receptor signaling** pathway				
gene_12290	prostaglandin G/H synthase 2-like	down	-1.193716184	9.20E-13
gene_21978	NF-kappa-B inhibitor alpha	up	1.127681941	3.95E-21
gene_8617	CD209 antigen-like protein D isoform X1	down	-1.187651252	5.22E-05
gene_13832	interleukin-1 beta-like	down	-1.868913751	0.000150372
**Complement and coagulation cascades**				
gene_17998	coagulation factor VIII-like	down	-2.508584976	2.68E-16

Table S4. Characteristics of 16S rRNA sequences of yellow catfish (*Pelteobagrus fulvidraco*) gills. C: 0 mg/L copper sulphate; T: 0.7 mg/L copper sulphate.

Samples	Raw Reads	Clean Reads	Denoised Reads	Merged Reads	Non-chimeric Reads
C1	80,067	79,918	79,538	77,668	72,970
C2	80,214	80,062	79,921	78,761	73,980
C3	80,532	80,380	80,260	79,913	73,190
C4	79,736	79,578	79,226	77,686	71,999
C5	79,889	79,732	79,663	79,386	74,687
T1	80,102	79,964	79,867	79,594	76,765
T2	80,042	79,915	79,783	79,048	76,427
T3	80,083	79,955	79,849	79,560	75,088
T4	80,295	80,165	80,078	79,289	75,561
T5	79,907	79,777	79,446	77,878	73,810

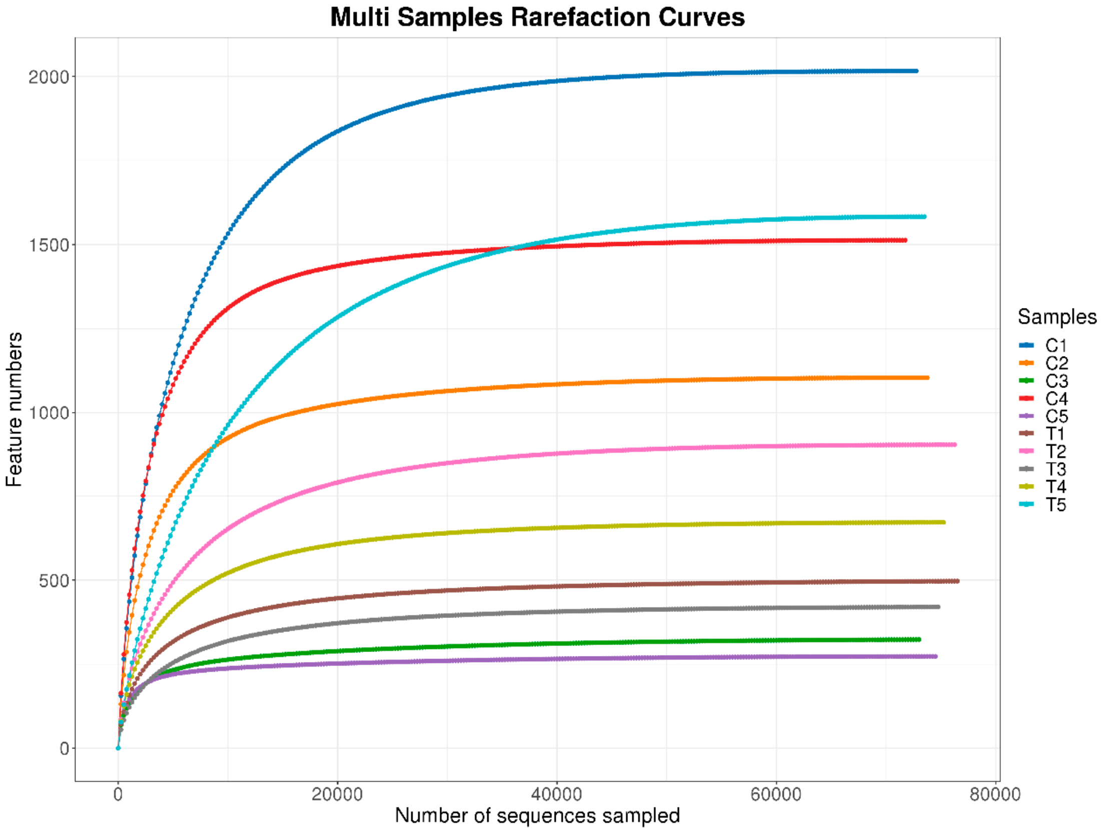

Figure S1. Rarefaction curves of yellow catfish (*Pelteobagrus fulvidraco*) gills tissue samples. C1-C5: control group; T1-T5: 0.7 mg/L copper sulphate exposed group.

Table S5. Richness and diversity indices of bacterial communities for all gill tissue samples. C: 0 mg/L copper sulphate; T: 0.7 mg/L copper sulphate.

Sample	Feature	ACE	Chao1	Simpson	Shannon	PD whole tree	Coverage
C1	2,016	2,016.1577	2,016.0	0.9876	8.6262	176.7335	1.0
C2	1,104	1,104.2607	1,104.0	0.9806	7.6941	94.3179	1.0
C3	323	325.9569	323.6176	0.9575	5.6062	58.6491	0.9999
C4	1,513	1,513.2642	1,513.0	0.9906	8.76	137.0542	1.0
C5	273	273.9218	273.037	0.9608	5.9133	45.1908	1.0
T1	497	499.8154	498.5	0.8734	5.1443	56.8091	0.9999
T2	903	903.6283	903.0484	0.9142	5.8949	88.8075	1.0
T3	420	422.0979	420.9032	0.7938	4.2062	52.0025	0.9999
T4	672	673.0993	672.25	0.8675	5.3941	77.8169	0.9999
T5	1,583	1,583.4281	1,583.0056	0.8344	5.3253	130.5898	1.0

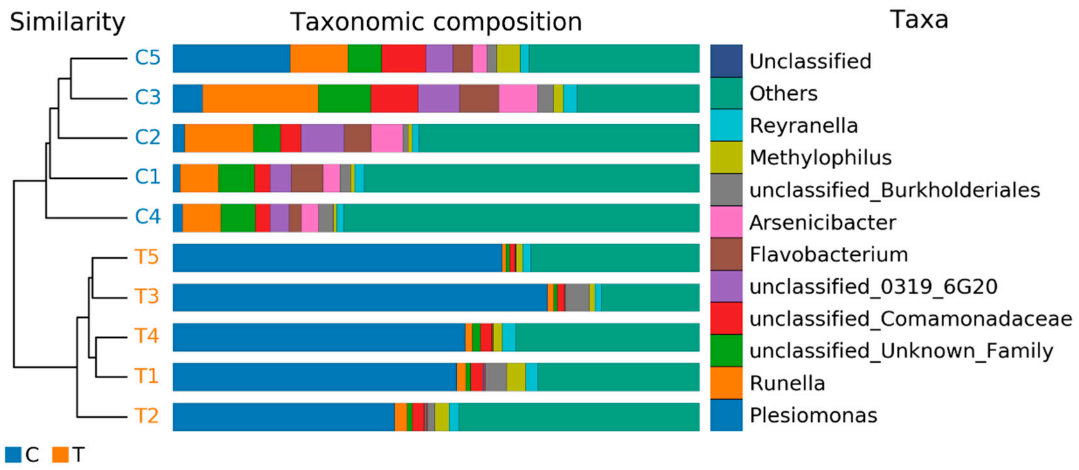

Figure S2. (Left) Hierarchical cluster analysis based on the unweighted pair group method with arithmetic mean (UPGMA); (Right) Relative abundances of dominant microbial genera. C: copper sulphate; T: 0.7 mg/L copper sulphate.

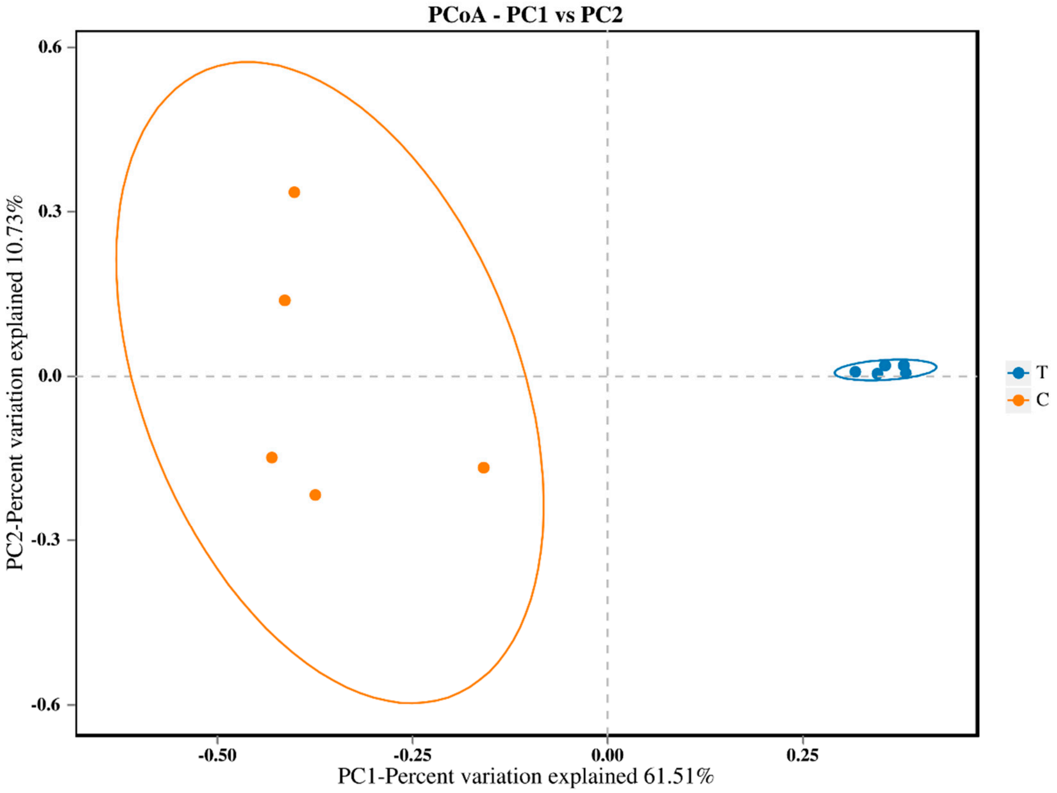

Figure S3. Principal coordinates analysis (PCoA) of the microbial communities. C: 0 mg/L copper sulphate; T: 0.7 mg/L copper sulphate.
